# Development of ‘Twazon’: An Arabic App for Weight Loss

**DOI:** 10.2196/resprot.5497

**Published:** 2016-05-16

**Authors:** Aroub Alnasser, Arjuna Sathiaseelan, Abdulrahman Al-Khalifa, Debbi Marais

**Affiliations:** ^1^ Food Science and Nutrition, King Saud University, SA, Applied Health Sciences, University of Aberdeen, Food Science and Nutrition, Applied Health Sciences, King Saud University, University of Aberdeen. Riyadh Saudi Arabia; ^2^ The Computer Laboratory University of Cambridge Cambridge United Kingdom; ^3^ King Saud University Riyadh Saudi Arabia; ^4^ Applied Health Sciences, University of Aberdeen Applied Health Sciences, University of Aberdeen Aberdeen United Kingdom

**Keywords:** weight loss, smartphone, mobile apps, Arabic, obesity

## Abstract

**Background:**

Weight gain and its related illnesses have become a major public health issue across the world, with Saudi Arabia and other Gulf countries seeing dramatic increases in obesity and overweight, and yet there is very little information on how to intervene with this demographic due to cultural and linguistic barriers. As the use of smartphones and apps has also increased in the region, information communication technologies could be a cost-effective means of facilitating the delivery of behavior-modification interventions directly to the target population. Although there are existing apps that offer lifestyle-modification tools, they do not give consideration to the evidence-based practices for weight management. This offers an opportunity to create an Arabic language weight loss app that offers localized content and adheres to evidence-informed practices that are needed for effective weight loss.

**Objective:**

This paper describes the process of developing an Arabic weight loss app designed to facilitate the modification of key nutritional and physical activity behaviors among Saudi adults, while taking into consideration cultural norms.

**Methods:**

The development of the Twazon app involved: (1) reviewing all available Arabic weight loss apps and compared with evidence-based practices for weight loss, (2) conducting a qualitative study with overweight and obese Saudi women to ascertain their preferences, (3) selecting which behavioral change strategies and guidelines to be used in the app, (4) creating the Saudi Food Database, (5) deciding on graphic design for both iPhone operating system and Android platforms, including user interface, relational database, and programming code, and (6) testing the beta version of the app with health professionals and potential users.

**Results:**

The Twazon app took 23 months to develop and included the compilation of an original Saudi Food database. Eight subjects gave feedback regarding the content validity and usability of the app and its features during a pilot study. The predominant issue among the group was the lack of information explaining how to use the app. This has since been resolved through the implementation of a tutorial. No other changes were required to be made.

**Conclusions:**

Information communication technologies, such as smartphone apps, may be an effective tool for facilitating the modification of unhealthy lifestyle habits in Saudi; however, consideration must be given to the target population, cultural norms, and changing trends in the global market. The effectiveness of the app will be better determined during a 6-month intervention with 200 overweight and obese Saudi women.

## Introduction

Obesity and overweight in the Saudi adult population has increased dramatically for both men and women, respectively; however, due to the cultural limitations and inaccessibility to physical activity, there is a predominant effect on the female population. Recent studies show that the rate of obesity and overweight in women has increased from 26.6% in the mid-1990’s to 33.3% in a national survey conducted in 2013 [1,2]. This is supported by the World Health Organization’s report published in 2014, which indicates that obesity has increased worldwide by more than 50% since 1980. This worldwide increase, paired with the statistical increase in Gulf countries, makes the prevalence of obesity a major public health issue in Saudi Arabia [3,4].

Certain local issues have contributed to the current and projected increase in the prevalence of overweight and obesity. These include aspects such as climate making it substantially harder to be physically fit [5], and the growing popularity of western fast foods resulting in a change in diet [6]. Additionally, the lack of nutritional and proportional information for regional dishes makes it difficult for locals to identify caloric content of meals [7]. Furthermore, cultural aspects relating to the status of women may also contribute to the higher prevalence of overweight and obesity in women specifically. These include high birth rate, and cultural restrictions that require women to stay inside the home, be accompanied by a male to go outside, and seek permission from family members to engage in physical activity [8]. Currently, the most popular method to lose weight among Saudi is gastric and bariatric surgery, as it is considered to be the fastest and most effortless method regardless of the risks associated with it [9].

Smartphone and app usage has shown exponential growth in Saudi Arabia in recent years ranking it third in smartphone usage in the world [10], with a penetration rate of 73% as of 2014 [11]. Saudi Arabia also has the highest-ranking Twitter usage in the world [12]. Cultural restrictions make it easier, for women especially, to express themselves socially and publically in a virtual environment through the use of smartphone technology. There is evidence that women are using websites and apps such as Instagram, Facebook, and Twitter to initiate home-based businesses [13] and participate in social solidarity [14].

The increasing ubiquity of these technologies therefore created an opportunity for the development of an Arabic app to be used as an appropriate tool to treat and prevent obesity in this population. To inform this development, two important aspects were investigated. First, Saudi Arabian women were consulted about their use and needs for a weight loss app [15]. Although women in Saudi Arabia reported using weight loss apps, they highlighted language barriers and cultural insensitivity, making recommendations for the development of a culturally sensitive Arabic weight loss app [15]. Second, it was important to ascertain whether there were any effective weight loss apps already available, specifically Arabic apps as recommended by the Saudi women. Although there was evidence available that English apps [16,17] fail to comply with evidence-informed practices for weight management, there was a paucity of evidence for Arabic apps. Screening of Arabic weight loss apps confirmed that they also did not comply with evidence-informed practices for weight management [18]. The development of an Arabic weight loss app including evidence-informed practices for weight management was therefore justified.

This article sets out to elaborate on the steps taken in the development of the Twazon app and tools included, the challenges that were faced, and the insights gleaned from the process.

## Methods

### Description of ‘Twazon’

The Arabic name, Twazon, means balance, which refers to the balance of dietary intake compared with energy expended through physical activity. The name and logo were developed through consultation with the community, including participants from the initial focus group discussions.

The use of the app begins with membership and social networking features. The user is required to complete a “5-step” registration process that allows them to use their email, Facebook, or Twitter accounts when signing in or out of their Twazon account. The process begins with the input of basic, required information such as name/nickname, gender, age, height, current weight, waist circumference, current health status, pre-existing diseases, and physical activity status. After submitting this information, the app provides the user with their ideal weight goal and the date by which this weight can be achieved by reducing the user’s daily caloric intake by 600 [19]. An optional tutorial presents the user with instructions on how to use the app.

There are many different theories that guide health promotion interventions. The social cognitive theory [20] forms the theoretical base of this application as it considers the importance of the social system in relation to the behavior of the individual, as well at the value of self-efficacy and self-regulation. It is considered a dynamic interaction between personal factors, behavior, and environment and confirms the importance of observational learning, which is based on observing others’ experience or results [20].

The Twazon app includes various tools to address evidence-informed practices for weight loss interventions as identified and used for screening of Arabic apps [16] (Table 1), and based on recommendations provided during the focus group discussions with overweight and obese Saudi women [15].

**Table 1:** Tools in Twazon addressing evidence-informed weight loss practices.

Practice	App Information
Weight assessment and goal setting	Assesses weight by calculating body mass index and waist circumference
	Allows users to set their ideal weight, and sets a target date for achieving the weight loss goal
	Calculates the number of calories needed daily based on their target weight
	Recommends a decrease of at least 600 calories consumed per day in order to achieve weight loss goal
Healthy diet	Recommends daily servings/portions of all foods and beverages, including 6 cups of water per day
	Recommendations given according to healthy lifestyle self-assessment score
	Provides a customized healthy food palm based on the user’s intake report
	Recommends the reading of labels and describes how to properly read labels
	Offers some suggestions for healthy food options in place of unhealthy food items.
	Allows users to correct a poor meal/diet as an education tool for menu planning
	Tips will be sent if the intake/activity ratio is off-balance according to healthy lifestyle self-assessment score
Physical activity	Recommends a minimum of 30 minutes of physical activity three times a week and allows users to assess their physical activity every 2 weeks
	Tips will be sent if the amount of physical activity is low
	Recommends taking at least 10,000 steps and provides a pedometer that tracks the daily number of steps
Self-monitoring	Allows users to track their daily food (calories) and water intake, and number of servings per food group, every 2 weeks
	Allows users to self-assess their physical activity, every 2 weeks
	Provides a weight loss tracker that informs user of current weight loss toward their goal weight (kg)
Social support	Provides an app-specific message board allowing users to privately share experiences, weight loss goals achieved, and photos with other users
	Allows users access to social networking services such as Twitter

### Evidence-Informed Weight Loss Practices

#### Weight Assessment and Goal Setting

Users will be able to assess their current weight goals by providing physical and lifestyle information. The app calculates the current body mass index and ideal body weight based on the formula from Lemmens et al [21]. The app will provide a realistic goals setting feature with a target weight loss of 0.5 to 1 kg (1-2 lb) a week [19] and will encourage modest loss of initial weight (5%-10%) as it is significantly correlated with meaningful changes in chronic disease risk [22]. The app will calculate the duration (days) required for the weight loss, enabling users to set appropriate and realistic goals. The app will also provide a daily caloric intake goal by calculating the daily calories needed based on the ideal weight with a recommended daily decrease of 600 calories less than current consumption.

#### Healthy Diet

**Figure 1:**
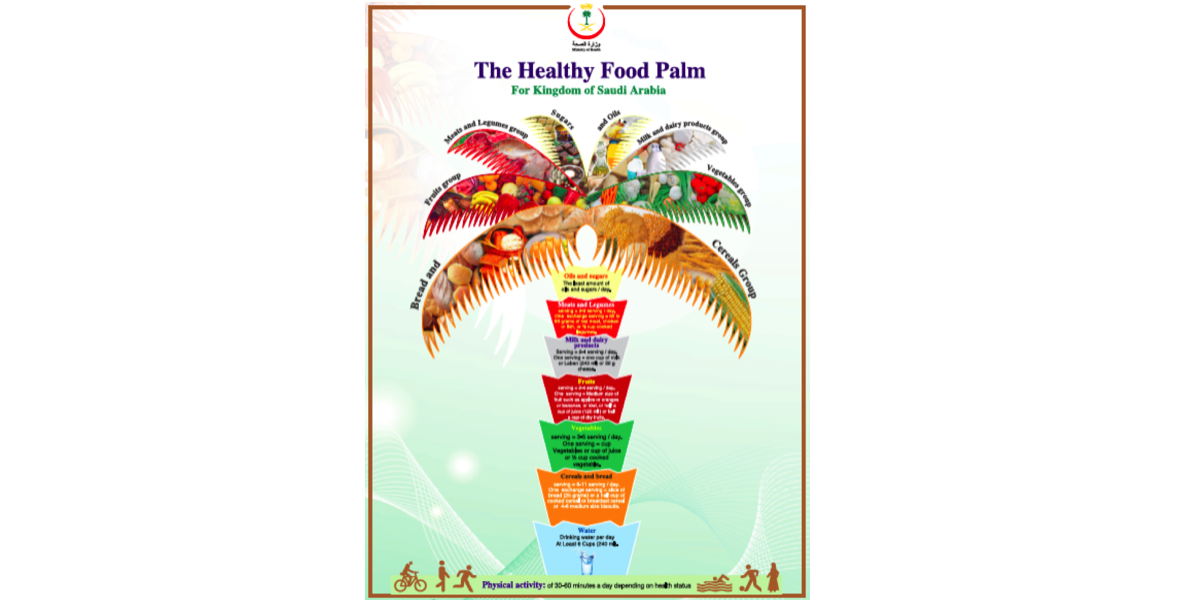
The Healthy Food Palm.

The app also includes a healthy lifestyle self-assessment score. Users will be prompted to complete the self-assessment every 2 weeks to track their level of achievement. As several studies have shown that there is a link between increasing consumption of a Mediterranean diet and lowering obesity rate [23,24], this self-assessment tool was developed using the Mediterranean diet assessment tool [25], however the alcohol question was excluded as alcohol intake is forbidden in the Saudi culture. Four additional questions were included based on the Saudi Healthy Food Palm guide [26] (Figure 1).

The additional questions cover aspects of dairy and whole grain consumption as well as daily physical activity. The healthy lifestyle self-assessment is therefore scored out of 17 and each question is linked to the relevant part of the Healthy Food Palm. Each question asks for the level of intake of a specific food/food group and this response is then used to calculate whether this is within the recommended levels or not (Textbox 1).

**Lifestyle self-assessment questions**

Vegetables:

- Are ≥2 servings (of 200 g each) of vegetables eaten each day?
- Are pasta, vegetable, or rice dishes flavored with garlic, tomato, leek, or onion eaten ≥2 a week?

Fruits:

- Are ≥3 servings of fruit (of 80 g each) eaten each day?

Oils:

- Is olive oil the main culinary fat used?
- Are ≥4 tablespoons of olive oil used each day?
- Is <1 serving (12 g) of butter, margarine, or cream eaten each day?

Sugar:

- Is <1 serving (330 ml) of sweet or sugar sweetened carbonated beverages consumed each day?
- Is <3 servings of commercial sweets/pastries eaten each week?

Meat and beans:

- Is <1 serving (100-150 g) of red meat/hamburgers/other meat products eaten each day?
- Are ≥3 servings (of 150 g) of legumes consumed each week?
- Are ≥3 servings of fish (100-150 g) or seafood (200 g) eaten each week?
- Is ≥1 serving (of 30 g) of nuts consumed each week?
- Is chicken, turkey, or rabbit routinely eaten instead of veal, hamburger, or sausage?

In addition to the 14-Item Mediterranean Diet Assessment above, these questions were added to the current assessment:

Dairy:

- Are ≥2 servings of dairy products consumed each day? (One serving=one cup of milk or yogurt, three slices of processed cheese slices)
- Are low fat or skimmed milk products consumed instead of full fat?

Bread and cereal:

- Are whole wheat grains consumed instead of refined grains?

Physical activities:

- Do you do physical activity for 30 minutes or more three times a week or more?

**Figure 2:**
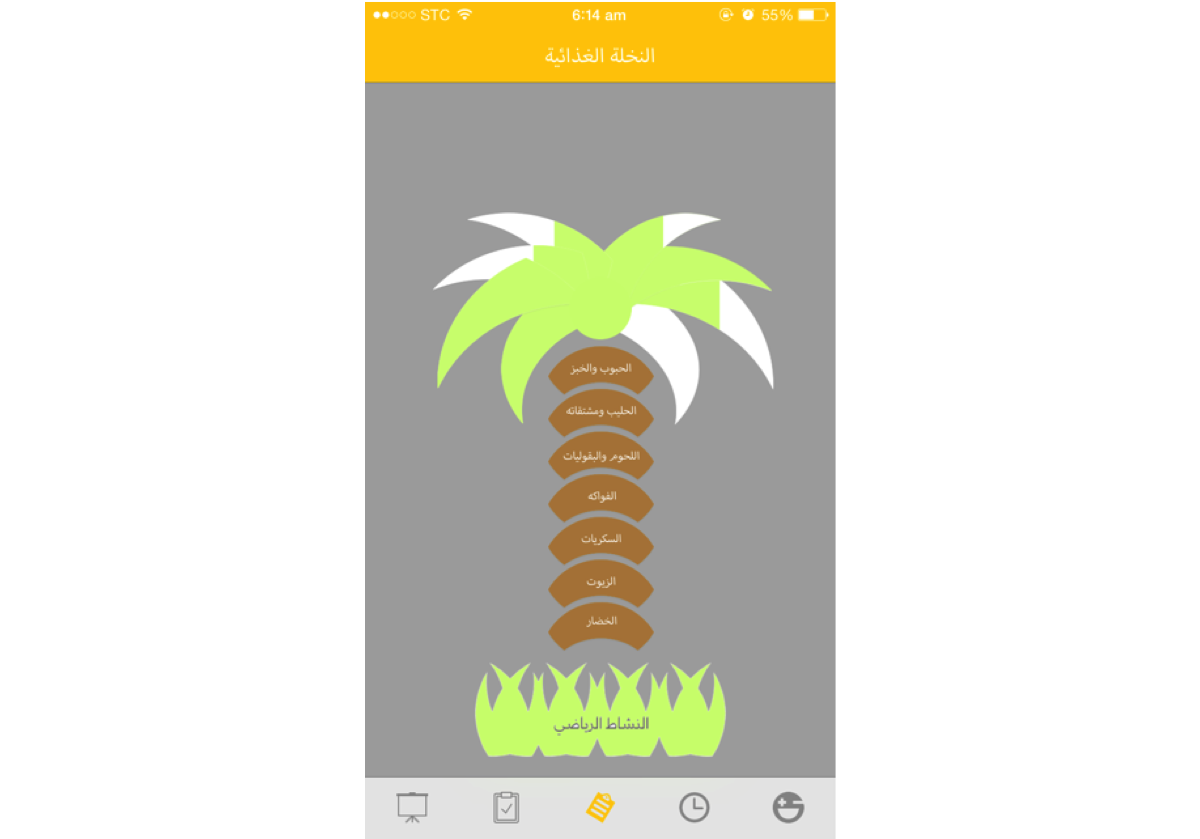
Graphic display of results of the healthy lifestyle self-assessment score.

The results of the assessment will be provided in a graphic format of the Healthy Food Palm guide and responses within recommended levels will turn the palm leaves green (Figure 2). For a full list of app prompts and feedback, see Supplementary Table 2.

**Figure 3:**
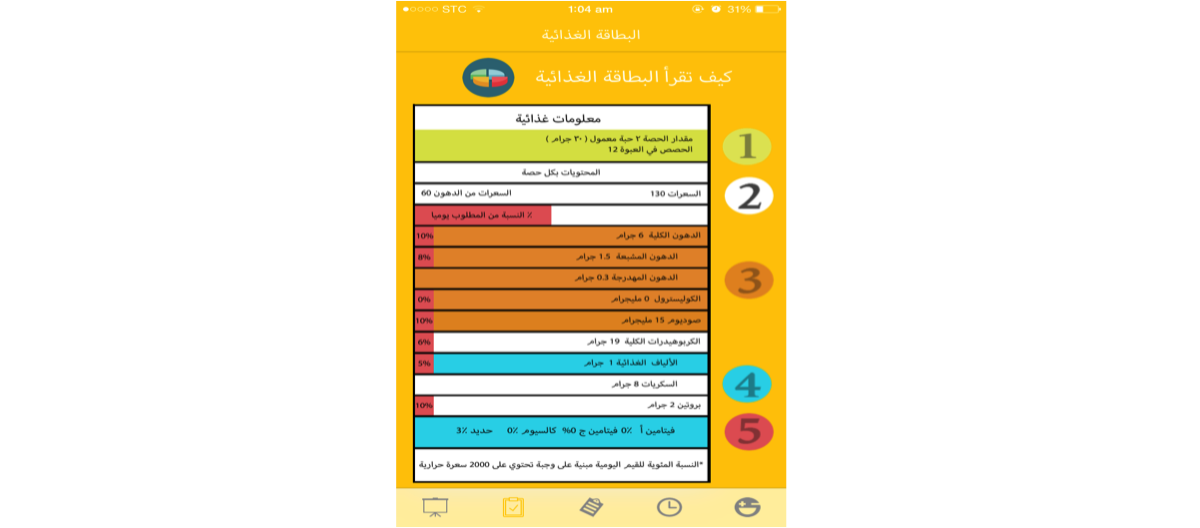
How to read food labels.

**Figure 4:**
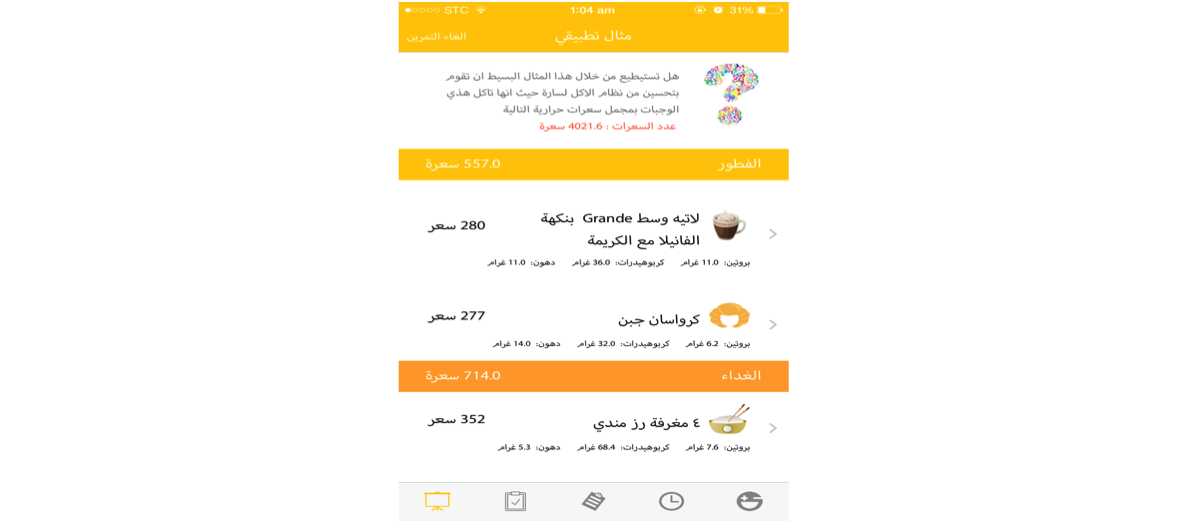
Education tool for menu planning.

A response below the recommended levels will prompt a notification to be sent to users with tailored tips based on the healthy food palm guidelines [26] as well as other international government health websites [27-29]. The app will recommend daily servings or portions of all foods and beverages, including six cups of water per day as well as describe how to properly read labels (Figure 3). The app will offer some suggestions for healthy food options in place of unhealthy food items and allows users to correct a poor meal/diet as an education tool for menu planning (Figure 4). Tips will be sent if the intake/activity ratio is off-balance according to the healthy lifestyle self-assessment score.

#### Physical Activity

Energy expenditure is calculated using the Metabolic Equivalent of Task values based on The Compendium of Physical Activities, which provides a wide variety of sports and fitness activities as well as activities of daily living [30]. The Twazon app physical activity practice was created to ensure context validity to include only activities that Saudi women would be involved in (H. Al-Hazza, email communication, July 2014). Physical activities that are traditionally not available to most women such as tennis, running, or playing sport outdoors have been replaced with suggestions to do exercises at home or find support from others in order to find physical activities that are easily accessible by Saudi women. The app will recommend a minimum of 30 minutes of a physical activity selected from a provided list, at least three times a week. The app will also recommend taking at least 10,000 steps per day and will provide a pedometer that tracks the daily number of steps. It will also allow users to assess their physical activity every 2 weeks, sending tips if the amount of physical activity is low.

#### Self-Monitoring

**Figure 5:**
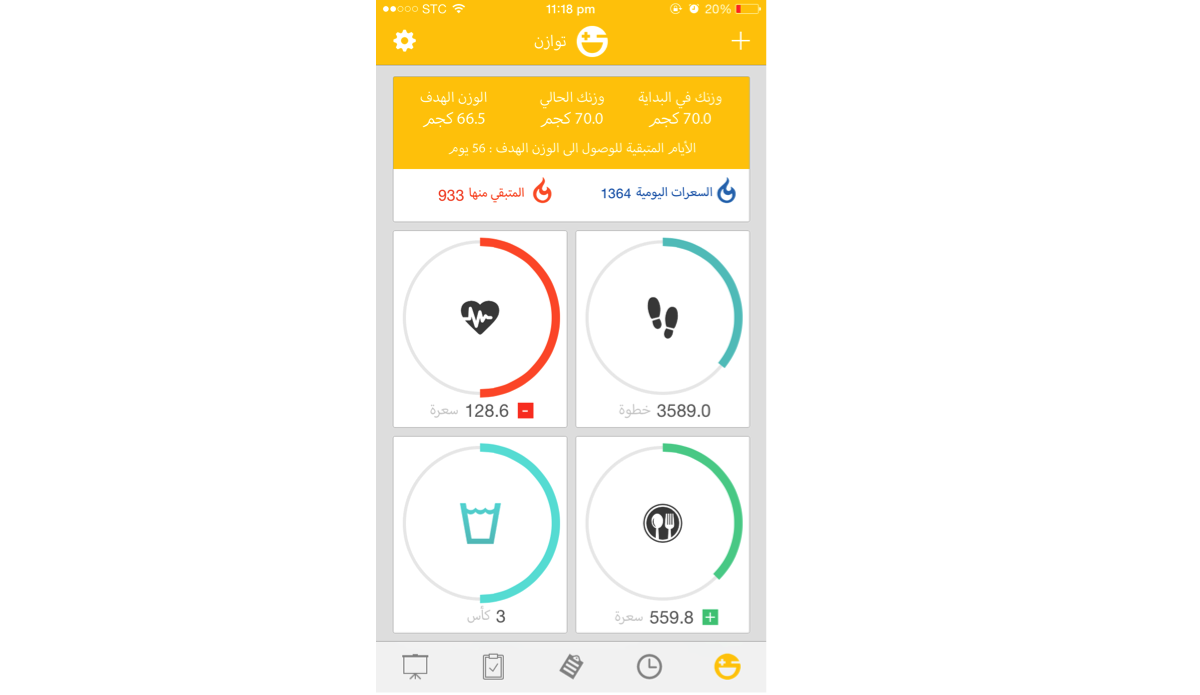
‘Twazon’ home page indicating daily self-monitoring of dietary intake and physical activity and goal tracking.

As there is a significant association between self-monitoring of both diet and physical activity and weight loss [31], the app will enable self-monitoring of daily energy (kCal) of food and drink consumed (in) and physical activity (out) as well as weight tracking. For the purposes of tracking the balance of calories (in vs out), the Twazon app includes customized databases for dietary intake (calories only) and physical activity. These databases will allow users to keep a daily food and physical activity diary. The home page, or dashboard, of the app will provide this information for easy access to tracking and goals (Figure 5).

The app will allow the users to monitor their weight progress including starting weight, current weight, and goal weight. The app allows the user to report the foods consumed daily and then calculates the equivalent amount of calories, providing the user with a number of calories remaining for the day. The dashboard will provide a system to track calories consumed, steps taken, water consumed, and physical activity. When a user exceeds the number of calories required in a day for weight loss, consumes more servings of a particular food group, or does not practice the minimum amount of daily physical activity, they will receive a notification informing them to engage in more physical activity.

#### Social Support

Twazon will encourage the user to connect to social networks to add social support, which has a positive effect on health behavior [32]. This allows participants to view other app users and their progress, post pictures, and share them on a variety of social networking services (eg, Instagram, Facebook, and Twitter) to facilitate real-time communication and peer support among participants. This was one of the major shortcomings identified in both English [16,17] and Arabic weight loss apps [18], and an aspect highly recommended for inclusion by overweight Saudi women during the focus group discussions [15].

#### Customized Saudi Food Database

A Saudi food composition database was developed to provide the caloric information of more than 400-food item servings. This database was developed specifically for the app and compiled using multiple data sources. These include the Saudi food composition tables [33], the food composition tables for the Kingdom of Bahrain [34], the Kuwait food composition tables [35], the US food composition tables (ESHA Food Processor, version 10.8, nutrient analysis software), and the UK food composition tables [36]. If the calorie value was not available in the Saudi food composition tables, the international food tables were consulted in a relevant order. In the event that there was a complete lack of food composition data for specific traditional dishes (eg, falafel sandwich, yogurt cucumber salad), common recipes taken from restaurants were added by calculating the nutrient values of ingredients using the ESHA food processor program. As the current Arabic food databases (Saudi, Bahrain, and Kuwait) measure food only in grams, average household measure portion sizes (cups, spoons) were calculated for each of the food items/dishes for which this information was not available. If a user consumes a product that is not found in the finished database, they are able to input the food item into a private database, which sends a notification to the administrator alerting them to update the database with the new product.

#### Behavior Change Techniques

All system requirements were based on proposed features for and ideal weight loss app by the target group [15] as well as trying to include all aspects for evidence informed practices. As most weight loss apps tend to lack behavior change techniques (BCT), these were incorporated into this app using Michie's taxonomy [37] to code the techniques (Supplementary table 2 in Table 2). A total of 29 BCTs were included, related to goals and planning (4 codes), feedback and monitoring (4 codes), social support (3 codes), shaping knowledge (1 code), natural consequences (1 codes), comparison of behavior (2 codes), associations (2 codes), comparison of outcome (1 code), repetition and substitution (3 codes), comparison of outcomes (1 code), rewards and threats (1 code), regulation (1 code), antecedents (3 codes), and identity (1 code). An overview of the system requirements, BCTs, and associated features in Twazon App is provided in Supplementary Table 2.

## Results

### Testing the App

The Twazon app was piloted as a preliminary evaluation by two groups made up of experts in the field and potential users. These groups were asked to use the app for 5 to 7 days and give feedback and suggestions.

#### Expert Testing

The group of Saudi health professionals included two physical activity specialists and three nutritionists/dietitians. They provided content validity by reading app info, tips, and goals set by the app, and so on, and verified that it was accurate based on their professional knowledge. They were given access to the app itself, as well as an attached document listing the app’s content sent by email. Three professionals reviewed the app and reported that it met all necessary criteria and found the content to be valid and accurate. They approved the information given to potential users although they raised a few questions about the use of the palm tree as the food guide, using waist-to-hip ratio instead of waist circumference, the accuracy of the “energy-expenditure formula,” and inquired about the relationship between the pedometer and the offline function.

#### Potential Users Testing

The group of potential users included 10 overweight Saudi women over the age of 18. They were asked to input their personal data into the app, and then follow the goals set by the app including any physical activity recommended. They checked for usability, design satisfaction, and any problems in the app’s system. In order to facilitate fast response times, their feedback was sent by WhatsApp. Five women submitted feedback on what they liked and disliked about the app. The majority of the users liked that the app raised awareness, and encouraged commitment and self-monitoring.

...It made me realize that a lot of foods that I eat between meals, which I don’t care about, have a great effect on increasing the calories.”

They also liked that it assigned a weight goal and calculated daily caloric intake, water consumption, and effort exerted.

“What I liked in the application is that it calculates my steps, and water quantity, and how many calories I consumed; even the effort; it could calculate it.”

The Healthy Food Palm Tree was also beneficial to the users by identifying current health status.

I liked the palm tree…I came to know my nutrition disorder through it unfortunately, and the need to adapt my eating and nutrition habits.”

The app’s design and simplicity was also well received and the users enjoyed being able to add specific dishes as “favorites” and reference them in the future.

### Future Suggestions

Suggestions made included that they would like to be able to view the history of the previous day’s food intake and activity, in addition to being able to “add forgotten meals” or activity. Moreover, the majority felt that the app needed to be even easier and should offer a detailed explanation on how to use the app. A couple of the women wanted notifications on meal times and one woman mentioned “the need for the caloric information for certain fruits and juices.” The results show that the usability of the app varies between potential users and this might be attributed to the different learning styles or technological literacy of users.

## Discussion

### Principal Findings

To our knowledge the development of previous Arabic weight loss apps has not complied with sufficient evidence-informed practices for weight management. The Twazon app differs from previous commercial Arabic weight loss apps in the following key areas: (1) it has been designed in collaboration with final users, (2) it complies with evidence-informed weight loss practices, (3) it provides social-network access, and (4) it includes a caloric content of Saudi local food.

The Twazon app, developed under the Android and iPhone operating system platforms, is a user-friendly, interactive app designed to track daily physical activity and food intake, and then provides customized advice to losing weight. The app took 23 months to build primarily because of the lack of information regarding household measurements of local foods [7]. During the creation of the local food databases, we reviewed the content of Arabic weight control apps and explored overweight Saudi women’s opinions in order to design a smartphone app to fit their needs.

The Twazon app was tested in collaboration with the local community and health professionals. Three health professionals determined content validity and five potential users (overweight Saudi females) evaluated the app for usability.

Overall, the app was reviewed to provide accurate information and was reported to have helped several of the potential users identify and understand their weight loss goals. The app was found to be aesthetically pleasing in design and many of the features were user-friendly.

In order to better explain the functions of the app and its features, a tutorial that includes pop-up messages will guide the user through the general use of the app, identifying the most important points and steps. The lack of nutritional data for certain foods will be solved by a detailed explanation of use in the aforementioned tutorial as well as a constantly updated crowd-sourced admin-reviewed food data bank. The users will be allowed to input their favorite daily foods, and an administrative team will review these foods to see if they should be added to the official data bank. Notifications of mealtimes and the ability to view and modify your “history” are features that are currently being worked on, and will be made available to users in the future.

### Limitations

The Twazon app possesses some limitations such as: (1) it doesn’t record the daily calories or activity into a history or backlog, (2) there is no meal planning available or recipes offered, (3) there is no professional support, (4) there is no bar-code scanning feature available as of yet, (5) it cannot generate new tips 365 days a year, and (6) for clarification, the app cannot work offline. Finding solutions to these limitations and upgrading the app consistently will be necessary to encourage and maintain engagement.

### Strengths

The Twazon app is different from other available weight loss applications in that it applies the information found in evidence informed practices and focus group findings, allowing for the specific needs of overweight Saudis to be met through a localized and tailored approach. The Twazon app is currently the only app to provide household measurements for local foods and dishes, making it easier for the user to manage their portion control. The app also offers recommended daily exercise that is culturally sensitive and suited to individual physical status. It also provides social networking features that allow users to connect and support each other. Based on the biweekly self-assessment, the users receive notifications that give tips about avoiding certain foods and increasing the intake of others.

### The Way Ahead

The effect of the app on weight loss will be examined in a 6-month pre-post intervention study with 200 overweight and obese women from Riyadh, Saudi Arabia. The primary endpoint will be the proportion of the group who lose at least 5% body weight.

## Appendix

Multimedia Appendix 1 Supplementary Table 1.

Multimedia Appendix 2 Supplementary Multimedia Appendix 2.

## Abbreviations

BCT: behavior change techniques
