# Supplementary material for: Development of ‘Twazon’: An Arabic App for Weight Loss
Source: JMIR Res Protoc. 2016 May 16;5(2):e76. doi: 10.2196/resprot.5497 (PMC4886101; doi:10.2196/resprot.5497)
Supplement: Multimedia Appendix 1 [file resprot_v5i2e76_app1.pdf]

Supplementary Table 1: Prompts and Feedback In Twazon App

Category	Prompts and Feedback
**Physical Activity:**	
User´s daily physical activity gauge	**Visual Feedback:**<br>*Mainpage* - Physical activity feedback circular gauge completes itself when one hour of physical activity is performed.
**Step Counter:**	
User´s daily step counter gauge	**Visual Feedback:**<br>*Mainpage* - Daily step feedback circular gauge completes itself when user has taken 10,000 steps.
User has completed less than 5,000 steps	**Message Prompt:**<br>*Stepcounter page* - "No one can reach 10,000 on the first try. Start gradually and try to add 500 steps daily."
User has completed 5000 - 7499 steps	**Message Prompt:**<br>*Stepcounter page* - "Good Job! You have reached half way! Keep walking!"
User has completed 7500-9999 steps	**Message Prompt:**<br>*Stepcounter page* - "Excellent, you have almost reached your goal, just a short distance more!"
User has completed 10000- 12499 steps	**Message Prompt:**<br>*Stepcounter page* - "Congratulations! You have reached your daily Goal! Try to make it a daily habit!"
User has completed 12500 steps or more	**Message Prompt:**<br>*Stepcounter page* - "you are very active, you should be proud!"
**Water Intake:**	
User´s daily physical activity gauge	**Visual Feedback:**<br>*Mainpage* - Water consumption feedback circular gauge completes itself when user consumes 6 cups of water.
User does not drink daily recommended amount of water	**Visual Feedback:**<br>*Mainpage* - Daily water intake circle remains gray.
User has consumed daily recommended amount of water	**Visual Feedback:**<br>*Mainpage* - Daily water intake circle turns blue.
**Calorie Intake:**	
User´s daily calorie intake gauge	**Visual Feedback:**<br>*Mainpage* - Calculation of user´s daily calorie intake and remaining daily calorie intake displayed through number and daily calorie circle.<br><br>*Food database* - Calculation of user´s daily calorie intake and remaining daily calorie intake displayed.
User is 200 calories away from daily limit	**Visual Feedback:**<br>*Mainpage* - Daily calorie intake circle turns red.
Daily calorie intake has exceeded the limit	**Visual Feedback:**<br>*Mainpage* - Daily calorie intake circle turns red.<br><br>**Message Prompt:**<br>*Food database* - "You exceeded your daily intake. We care about your health"
User has consumed equal to or less than daily calorie limit	**Visual Feedback:**<br>*Mainpage* - Daily calorie intake circle is green.
**Goal Setting and Assessment:**	
Number of days user has to complete goals	**Visual Feedback:**<br>*Mainpage* - Counter counts down number of days until

weight loss goal is complete.

Remind users to adjust weight information so as to calculate weight loss goals every 2 weeks.

Message Prompt:
Mobile Device Notification - “Please update your weight information.”

Remind users to complete two week assessment in order to adjust which tailored health tips are displayed.

Message Prompt:
Mobile Device Notification - “Please update your palm tree information.”

Give user updated information about their BMI as they engage with the app.

Visual Feedback:
Sign-up page: BMI number with classification displayed (underweight, healthy weight, overweight, obese)
Settings page: BMI number with classification displayed (underweight, healthy weight, overweight, obese)

Demonstrate to user how to measure their waist, and give information regarding risk for developing chronic disease based on their results.

Visual Feedback:
Settings page: Photos demonstrating correct method of waist measurement are given with cue for user to enter their measurement into the app.

Message Prompt:
Setting page: User is notified with a message regarding risk for developing chronic disease based on their waist measurement.
Examples:

“Your waist circumference is more than 80cm, this increases the risk of developing chronic disease”

“Your waist measurement is more than 88cm, you have a greatly increased risk to develop chronic disease”

Health tips:

General health tips sent to all users (frequency based on users preference settings).

Message Prompts:
Mobile Device Notifications - General health tips are sent to users from a library of 26 health tips.

Examples Include:

“Use oven cooking instead of frying in oil or fat”

“There is no one food that contains all the known essential nutrients”

“Balance your diet during the day, if you eat a big meal at lunch, eat light one at dinner”.

Tailored health tips sent to users based on palm tree assessments.

Message Prompts:
Mobile Device Notifications - Tailored notifications are sent to users from a library of 115 health tips. Topics of tailored tips are advice on: Fruit, Milk, Meat, Bread and Cereals, Oils, Sugars and Fitness.

Examples Include:

“Use low fat dairy products rather than whole fat”

“When ordering food don't add extra cheese or mayonnaise”

“Popcorn is a light and healthy snack”

Social Notifications:

Notify user of new followers inside Twazon social network.

Message Prompt:
Mobile Device Notifications - “(name) started following you”

Notify user of new “like” on their post.

Message Prompt:
Mobile Device Notifications - “(name) likes your post”

Notify user of number of followers.

Visual Feedback:
Social Account Page - “(number) following you” / “you are

following (number)” / number of posts
