# Supplementary material for: Development of ‘Twazon’: An Arabic App for Weight Loss
Source: JMIR Res Protoc. 2016 May 16;5(2):e76. doi: 10.2196/resprot.5497 (PMC4886101; doi:10.2196/resprot.5497)
Supplement: Multimedia Appendix 2 [file resprot_v5i2e76_app2.pdf]

Supplementary Table 2: An overview of the system requirements, BCTs, and associated features in Twazon App

System requirements (a)	BCT (b)	The App
Arabic language and culturally sensitive.	**Shaping Knowledge:** 4.1 Advise or agree on how to perform the behaviour.	Provides all information in the Arabic language with localized content in a culturally sensitive manner, allowing for the users to understand advice on how to lose weight.
Motivational tools, positive reinforcement, professional advice, support and social networking.	**Social Comparison:** 6.2 Draw attention to others' performance to allow comparison with the person's own performance.	Motivation through social comparison is delivered through a social network in which users are able to see what percentage of their and other's goals have been met.
	**Information about Other´s Approval:** 6.3 Provide information about what other people think about the behavior.	Provides feedback and motivation to users through the social network regarding other´s perception of their posts. For example they can receive "likes" for posting what they consumed for breakfast.
	**Non-specific reward:** 10.3 Arrange delivery of a reward if and only if there has been effort and/or progress in performing the behavior	Messages which congratulate and motivate users appear on screen when step counter goals are achieved. Additionally a green palm tree is shown to users when all the necessary weight loss documentation is complete to meet their goals over a two week period.
	**Identification of self as role model:** 13.1 Inform that one's own behavior may be an example to others	Users are encouraged to consider themselves as role model for their children or family members.
	**Credible Source:** 9.1 Present verbal or visual communication from a credible source in favour of or against the behavior.	Positive reinforcement and professional advice is provided through the app by sending messages to the user encouraging them to continue to meet their goals and giving information about health. . App messages are considered as a credible source as it is an evidence based app.
	**Instruction on how to perform a behavior:** 4.1 Advise or agree on how to perform the behavior.	Professional advice for how to best apply evidence informed practices offered throughout all tips and notifications of the app.
	**Information about health consequences** 5.1 Provide information about health consequences of performing the behavior	Professional information about the health consequences of abdominal obesity and unhealthy food choices are provided to users.
		Advice is offered to users to support

	**Remove access to the reward**: 7.4 Advise or arrange for the person to be separated from situations in which unwanted behavior can be rewarded in order to reduce the behavior	them in making healthy choices that separate them from high calorie food on a regular basis. Eg. Don´t bring cupcakes to work, choose a healthy snack instead.
	**Behaviour Substitution:** 8.2 Prompt substitution of the unwanted behavior with a wanted or neutral behavior	Advice is offered to replace behaviours which are likely to contribute to obesity with healthier life choices.
	**Restructuring the Physical Environment:** 12.1 Change, or advise to change the physical environment in order to facilitate performance of the wanted behavior or create barriers to the unwanted behavior	Professional advice for how to restructure users´ living environment to support the ease of making healthy decisions is provided in the app.
	**Restructuring the Social Environment:** 12.2 Change, or advise to change the social environment in order to facilitate performance of the wanted behavior or create barriers to the unwanted behavior	App advises users to restructure their social environment through recommending connecting with others in the weight loss community, spending time playing with children, etc. in order to facilitate weight loss.
	**Habit Reversal:** 8.4 Prompt rehearsal and repetition of an alternative behavior to replace an unwanted habitual behavior	Suggestions are given to rehearse new healthy habits in place of unhealthy habitual behaviours.
	**Social Support (unspecified)** 3.1 : Advise on, arrange or provide social support or noncontingent praise or reward for performance of the behavior.	Provides an app-specific message board allowing users to share their experiences and provide one another with counselling and encouragement as they work towards their individual goals.
	**Social Support (practical):** 3.2 Advise on, arrange, or provide practical help for performance of the behaviour.	App allows users share practical weight-loss ideas with other users inside the app´s social network or through other social networks.
	**Social support (emotional):** 3.3 Advise on, arrange, or provide emotional social support for performance of the behaviour.	Message board and social network links are integrated to allow users to give and receive emotional support as they are able to privately share experiences, weight loss goals achieved, and photos with other users.
Dietary and physical activity tools	**Goal-setting behavior**: 1.1 Set or agree on a goal in terms of the	Weight-loss goals are established with users after entering personal

	behavior to be achieved	information to calculate BMI. Users are able to identify their ideal weight allowing the app to set a target date and calorie consumption strategy to reach it.
	**Goal-setting outcome:** 1.3 Set or agree on a goal defined in terms of a positive outcome of wanted behavior	Weight-loss goals of .5 Kg per week are set with users as an outcome of following app advice.
	**Action planning:** 1.4 Prompt detailed planning of performance of the behavior	Reminders are regularly sent to users to encourage the correct amount of steps taken, water consumption, and physical activity. A warning is also sent when total daily calories have been consumed encouraging users to stop eating.
	**Discrepancy between current behavior and goal:** 1.6 Draw attention to discrepancies between a person's current behavior and the person's previously set outcome goals, behavioral goals or action plans.	Visual features in App are displayed to users to demonstrate discrepancies between their current behaviors and the guidelines that should be followed to meet weight-loss goals.
	**Graded Tasks:** 8.7 Set easy-to-perform tasks, making them increasingly difficult, but achievable, until behavior is performed	Graded weight loss goals are included in the app as well as a graded step counter tool which increases the amount of steps over time.
	**Conserving Mental Resources:** 11.3 Advise on ways of minimising demands on mental resources to facilitate behavior change	Instructs users how to read nutritional labels to minimise the need to memorise which foods are healthy for them and make healthy choices regularly. Additionally provides an extensive food calorie guide which users can use as a quick reference for calorie counting.
	**Body changes: 12.6** Alter body structure, functioning or support directly to facilitate behavior change.	Physical activity is promoted throughout the App. Utilizing step counter to promote physical activity.
	**Reduce prompts/cues**: 7.3 Withdraw gradually prompts to perform the behavior	Prompt settings are adjusted according to the user´s reports on behaviour performance, reducing messages when behaviour is performed.
Tailored information and feedback	**Feedback on behaviour:** 2.2 Monitor and provide informative or evaluative feedback on performance of the behavior	Informative feedback is provided to users based on their daily and bi-weekly records..
	**Self monitoring of behaviour:** 2.3	Daily diary and bi-weekly records

	Establish a method for the person to monitor and record their behavior(s) as part of a behavior change strategy	are used to monitor and record behaviors.
	**Self monitoring of outcomes of behaviour:** 2.4 Establish a method for the person to monitor and record the outcome(s) of their behavior as part of a behavior change strategy	User is asked to weigh themselves every two weeks and record their weight to update application setting.
	**Feedback on outcomes of behaviour:** 2.7 Inform the person of how much weight they have lost following the implementation of a new exercise regime.	App gives information to users about how many calories are lost during various physical activities.
	**Reduce prompts/cues:** 7.3 Withdraw gradually prompts to perform the behaviour.	Feedback messages are adjusted after every 2 week assessment. Messages are tailored to individuals behavior and reduced when user has demonstrated success in performing the behaviour.
User-friendly interface.	**Shaping Knowledge:** 4.1 Advise or agree on how to perform the behaviour.	Behaviour Change Techniques have been integrated in the App with a user-friendly interface in order to assist users in understanding the necessary steps they must take in losing weight.

(a) What the app should provide, arrange or do based on the qualitative study: What Overweight Women Want From a Weight Loss App: A Qualitative Study on Arabic Women [15].

(b) BCTs as described and classified by Michie et al. [37].
